# Developmental changes of Reelin-expressing cell populations in the marginal zone of the neocortex of the European wild boar, *Sus scrofa*

**DOI:** 10.1007/s00429-025-02958-w

**Published:** 2025-06-11

**Authors:** Eric Sobierajski, Miriam González-Gómez, Emilio González-Arnay, Petra Wahle, Gundela Meyer

**Affiliations:** 1 Faculty of Biology and Biotechnology, Developmental Neurobiology, Ruhr University Bochum, 44870 Bochum, Germany; 2 Institute of Neurocience, University of La Laguna,, 38200 Santa Cruz de Tenerife, Tenerife Spain; 3 Department of Basic Medical Science, Faculty of Medicine, University of La Laguna, 38200 Santa Cruz de Tenerife, Tenerife Spain

**Keywords:** Reln, Tbr2, PCNA, βIV-spectrin-positive AIS, Glutamate decarboxylase, Interneurons

## Abstract

Cortical Cajal-Retzius cells (CRc) are the main source of Reelin and essential for radial migration. We studied the development of cells expressing Reelin, Tbr1, p73 and calretinin in pig neocortex from E35 to P30. CRc originated around E35 from the cortical hem and filled the marginal zone (MZ). At E45, in an expanding cortex, CRc were more loosely arranged and more mature. At midgestation E60-E70, CRc with elongated horizontally oriented somata and long dendrites formed a dense network of processes in the upper MZ. Their axons were characterized by the presence of long axon initial segments and densely ramified in the lower MZ. Concurrently, the first GAD-positive/Reelin-positive as well as GAD-positive/Reelin-negative interneurons appeared in the MZ and in cortical layers. At E85, in an already highly gyrated cortex, the switch in Reelin-positive neuronal populations was largely completed. At E100 and P30, CRc were rare. A subpial granular layer was not detectable in pig. In sum, by form, distribution and the presence of a calretinin-positive axon plexus in the lower MZ, the pig CRc closely resembled the lagomorph and carnivore rather than the human morphotypes, and they become complemented by interneurons already after midgestation.

**Supplementary Information:**

The online version contains supplementary material available at 10.1007/s00429-025-02958-w.

## Introduction

Cajal-Retzius cells (CRc) are a transient cell population of the marginal zone (MZ) (Cajal 1980; Cajal 1891; Retzius 1893, 1894). They are evolutionary preserved from reptile to human (Marín-Padilla, 1998; Meyer and Goffinet 1998; Tissir and Goffinet 2003; Pérez-García et al. 2001; Goffinet et al. 1999; Cabrera-Socorro et al. 2007). Their peculiar morphology and molecular profile have been the subject of numerous studies across species (reviewed e.g. by Meyer et. al, 1999; Martinez-Cerdeño and Noctor, 2014; Molnár et al. 2006; Kirischuk et al. 2014) most of which focused on rodent and human. A main origin of neocortical CRc is the cortical hem, a signaling center at the interface of the choroid plexus and the future hippocampus, from where they migrate tangentially over the neocortex (Grove et al. 1998; Meyer et al. 2002; Yoshida et al. 2006). Additional sources are septum, thalamic eminence, and the retrobulbar area (Meyer et al. 2002; Meyer and Wahle 1999; Bielle et al. 2005) which altogether comprise the “forebrain hem system” (Meyer 2010; Roy et al. 2014).

CRc roles in cortex development have been unraveled with genetically modified mice and in-vitro studies (reviewed by Causeret et al. 2021; Elorriaga et al. 2023; Moreau et al. 2023). CRc secrete the extracellular matrix protein Reelin (D'Arcangelo et al.^1995^; Ogawa et al. 1995) that initiates a signaling cascade via the lipoprotein receptors ApoER2 and VLDLR responsible for the laminar positioning of neurons through tyrosine phosphorylation of the adapter protein disabled 1 (Dab1), expressed by the radially migrating pyramidal cells from the ventricular zone (VZ) into the cortical plate (CP) (Hiesberger et al. 1999; Trommsdorff et al 1999; Rice et al. 1998). CRc express the transcription factors Tbr1, a marker of early-born pallial glutamatergic neurons (Hevner et al. 2003; del Río et al. 1995), and p73, a member of the tumor suppressor p53 family (Kaghad et al. 1997; Yang et al. 2000; Meyer et al. 2002). P73 is expressed both as a transactivation-competent isoform (TAp73), described to promote apoptosis, and as an N-truncated isoform (DeltaNp73), thought to be anti-apoptotic (Pozniak et al. 2000; Jacobs et al. 2004). CRc express both variants (Meyer et al. 2019; Tissir et al. 2009), and the equilibrium between both may contribute to their early survival and final death. The calcium-binding protein calretinin is another characteristic marker of mouse and human CRc (del Río et al. 1995; Alcantara et al. 1998, Abraham et al. 2005; Meyer and González-Gómez 2018a, b).

During development, the MZ is populated by later-appearing GABAergic Reelin-positive interneurons which occupy also the cortical layers (Schiffmann et al. 1997; Pesold et al. 1998; Perez-Garcia et al. 2001; Alcántara et al. 1998). Reelin in CRc and in interneurons has different though complementary roles in cortex development, with CRc and Reelin-positive interneurons cooperating in different aspects of migration and laminar positioning (Alcántara et al. 2006; Vilchez-Acosta et al. 2022).

In the fetal human cortex (Marín-Padilla, 1990), and similarly in macaque (Zecevic and Rakic, 2001), CRc are closely related to and even embedded in the subpial granular layer (SGL) of Ranke (Ranke 1910; Brun 1965). The SGL is a transient layer which also contains small GAD- and calretinin-expressing neurons (Zecevic and Rakic, 2001; Meyer and González-Gómez 2018a, b). An SGL has hitherto not been described in non-primate species. We examine here whether an SGL is present in the pig cortex. In human, cortical growth, surface expansion and gyration are on a much larger scale than in conventionally studied laboratory animals. Similarly, in the large, highly folded human cortex, CRc have more complex morphologies and developmental histories than their rodent counterparts (Meyer and González‐Hernández, 1993; Meyer and González-Gómez 2018a, b), which in part may be related to the human accelerated region (HAR) RNA gene HAR1F, specifically expressed in human CRc (Pollard et al. 2006). Large-brained animals such as the pig could inform on the role of CRc in cortical development and evolution. Pigs are highly intelligent and emotionally differentiated (Gieling et al. 2011; Marino and Colvin 2015). As ungulates, they are precocially born with an almost mature cerebral cortex (Ernst et al. 2018), and an autonomous somatomotor behavior at birth which allows e. g. for testing neonatal piglets on spatial learning tasks (Elmore et al. 2012). We previously found that the neuropeptide Y neuronal system, microglia cells, myelin proteins, as well as blood vessels and blood–brain barrier proteins develop rapidly prior to birth (Ernst et al. 2018; Sobierajski et al. 2022, 2023, 2024). Recently, we reported that the E100/E110 somatosensory cortex expresses functional neuronal markers at the same level as the P30 cortex, and further, the proportion of mushroom spines at E110 is statistically not different from the proportion at P30 (Sobierajski et al. 2025). Thus, cortical maturation proceeds rapidly in pig and much earlier than in altricial rodents and carnivores, in which neural differentiation proceeds largely after birth.

Since their body mass, organ size, physiology, and immune system strongly resemble those of humans, the pig is increasingly considered a translational model that may fill the gap between studies in small laboratory animals and clinical trials in humans and that can be used for organ transplantation (Carrier et al. 2022; Healey 2025). The developing pig brain may also help to understand the process of gyrification, which allows tangential expansion of the cortical surface maintaining the basic mechanisms of radial migration common to all mammals (Rakic 1995). A possible role of CRc in cortical folding was suggested in the perinatal human cortex, where late-appearing CRc were predominantly observed in smaller, secondary and tertiary sulci (Meyer and González-Gómez 2018a, b). Even in mice, CRc are necessary for the formation of the hippocampal fissure (Meyer et al. 2019). Interestingly, a peak of Reelin expression was detected in the fetal pig cortex (Nielsen et al. 2010) coincident with the period of most intense gyrification (Ernst et al. 2018). In this context, it is important to note that mutations of the human *REELIN* gene result in a profoundly abnormal, lissencephalic cortex associated with cerebellar hypoplasia and severe mental deficiency (Hong et al. 2000). Similarly, mutations resulting in a total loss of function of *TP73* in homozygous human patients also lead to lissencephaly with severe mental incapacitation (Wallmeier et al. 2021).

## Materials and methods

### Animal material

Our animal of choice is the European wild boar (*Sus scrofa*), which does not show the effects of domestication, such as a reduction of total brain weight by 41% (see Ernst et al. 2018; Sobierajski et al. 2024). The material was obtained from the Üfter Mark area, North Rhine Westfalia which is managed by the Regionalverband Ruhr Grün. Wild boar sows were individually hunted in accordance with the German Game Law (Jagdrecht) or killed in road accidents and examined by the chief forest ranger Mr. Christoph Beemelmans. Law requires the disposal of viscera including sexual organs. Dissection of the fetuses from pregnant sows followed by initial immersion fixation in cold 4% paraformaldehyde in 0.1 M phosphate buffer, pH 7.4 was done at the Forsthof Üfter Mark by Ms. Christa Beemelmans. Fine dissection of the brain and body organs was done at Ruhr-University Bochum. Staging was done according to crown-rump-length and external features (Henry 1968) and the expertise of the forest ranger. Details of the fetuses, sows, and postmortem times until dissection of fetal brains have been reported earlier (Ernst et al. 2018; Sobierajski et al. 2022).

### Human material

Human brains were from our collection (e.g. Meyer et al. 2000; González-Gómez and Meyer, 2014; Meyer and González-Gómez 2018a). Material was fixed in Bouin or Carnoy solution, embedded in paraffin, and cut at 10 μm thickness.

### Ethical Statement

Material has been obtained through tissue sharing. The human material is from the brain tissue collection at University La Laguna, Spain, and has been frequently used in numerous previous studies since 1993. Human brains were from legal abortions following national guidelines in Spain, under the supervision of the Ethical Committee of the University La Laguna, in accordance with the Declaration of Helsinki, 1964, and with informed consent from the parents.

Material is from the pig brain collection at Ruhr University Bochum and has been used in several recent studies. Animals have been road-killed or hunted solely for the reason of population control or for instance, minimize agricultural damage. All visceral organs including fetal material and also postnatal nervous tissue of wild hoofed animals must be discarded according to German Game Law because it is not allowed to enter the human food chain. This material has been donated to us by the Ruhrverband Grün and Üfter Mark chief forest ranger Mr. Christoph Beemelmans. Since the animals were not killed for scientific purpose, our studies did not require any official permit.

### Immunostaining and antibodies

The human material was stained as described (Meyer et al. 2000; Meyer and González-Gómez 2018a). After an initial fixation the pig brains for paraffin embedding were postfixed in Bouin fixative for 2 days, dehydrated and embedded in paraffin following standard procedures. The E35 and E45 brains were processed with the skull left intact. Brains of the older stages were dissected from the skull, and in contrast to the pig material used in our parallel studies involving cryostat sectioning, the meninges were not removed. This was done to preserve the integrity of the MZ/layer 1, although it sometimes led to unspecific staining or reaction of endogenous peroxidase in blood cells and vessels. The tissue was cut into 10–15 μm thick coronal sections. Sections were deparaffinized, hydrated, and boiled in 10 mM citrate buffer (pH 6) for 20 min for antigen retrieval, rinsed in Tris-buffered saline (TBS, pH 7.6, 0.05 M), and incubated in the primary antibodies overnight in a humid chamber. After rinsing, they were incubated in the corresponding biotinylated secondary antibodies (rabbit anti-mouse IgG or goat anti-rabbit IgG; Dako, Glostrup, Denmark), diluted at 1:200 in TBS, followed by incubation with avidin–biotin complex (ABC, DAKO, Glostrup, Denmark) in TBS. Bound peroxidase was revealed with 0.04% 3,3-diaminobenzidine (Sigma-Aldrich, USA), 0.05% ammonium nickel (II) sulfate, and 0.03% hydrogen peroxide in TBS pH 7.6. Sections were dehydrated, cleared, and coverslipped using Eukitt® (O. Kindler, Freiburg, Germany).

The following primary antibodies were used: a polyclonal antibody anti-p73α (against amino acids (aa) 427–636 of full length p73, 1:300, gift of D. Caput); monoclonal anti-Reelin antibody 142, raised against aa 164–405 (gift of A.M. Goffinet); rabbit polyclonal against glutamate decarboxylase (GAD 65/67) (ab49832, 1:1000, Abcam); mouse monoclonal anti-PCNA (proliferating cell nuclear antigen) (PC10, ab29, 1:500, Abcam); rabbit polyclonal Tbr1 antibody (AB2261, 1:100, Millipore); rabbit polyclonal anti-Eomes antibody (antibody HPA028896, 1:100, Sigma-Aldrich), mouse monoclonal anti-Vimentin antibody (ab 20,346, 1:200, Abcam). For double-immunofluorescence on paraffin sections, antibodies mouse anti-Reelin 142 rabbit anti-GAD were mixed and incubated overnight at room temperature. The secondary biotinylated goat anti-mouse IgG, H&L chain specific biotin conjugate (cat. no. 401213, 1:300, Calbiochem) and Alexa Fluor 488 goat anti-rabbit IgG (H + L) antibodies (cat. no. A11001, 1:300, Invitrogen) were incubated for one hour at room temperature in the dark, followed by incubation with streptavidin Cy3 conjugate (cat. no. SA1010, 1:500, Invitrogen) for one hour at room temperature in the dark. DAPI staining for nuclei turned out too weak for being useful, possibly due to strong Bouin fixation. Sections were then rinsed in PBS and coverslipped with DABCO (1%) and glycerol-PBS (1:1). Slide-mounted 30 µm thick cryostat sections of E70 cortex were used for single and double-fluorescence staining (Sobierajski et al. 2022, 2024) with guinea pig polyclonal anti-calretinin (1:300; cat. no. 214104, Synaptic Systems) and rabbit polyclonal anti βIV-spectrin (1;400, gift of M. Engelhardt; Gutzmann et al. 2014) followed by appropriate secondaries (donkey anti-guinea pig Alexa-594, Dianova, Cat# 706–757-148, 1:1000; and donkey anti-rabbit Alexa-488, Thermo Scientific, Waltham MA, USA, RRID: AB_2687506, 1:1000). Similarly, we double-stained cryostat sections of 50 µm thickness with calretinin and tomato lectin, a well-established marker for blood vessels in normal tissue (Battistella et al. 2021). Negative controls were performed by omitting the primary antibodies.

### Analysis

Photomicrographs were taken with a Zeiss microscope and arranged. Fluorescent stainings were imaged using a Olympus Fluoview 1000 or a Leica TSC SP5 laser scanning confocal microscope (10 × and 40 × objective with 1.1 NA, 1024 × 1024 px). Global whole-picture contrast-, brightness-, color intensity- and saturation-settings were adjusted with Adobe Photoshop®. To document position and density in MZ/L1, Reelin-positive cells were plotted with the Neurolucida system (MicroBrightField, Inc., Vermont, USA). For a quantitative assessment, Reelin-positive cells were determined with the Neurolucida system in 25 µm bins (at E35 and E45) and 50 µm bins (from E60 onwards) from the pial surface in the MZ, and also in 100 µm bins in the forming layers down to the L6/white matter border. A potential association between CRc axonal boutons and blood vessels was tested as follows. Confocal stacks of the calretinin-positive axon plexus containing tomato lectin-positive blood vessels in the lower half of the MZ were taken. After deconvolution, a 3D-structure for the blood vessels was reconstructed (Imaris). Spherical spots were generated (Imaris) for every CR + bouton-like punctum located around the blood vessel of interest. A filter allowed to exclude any spots with a surface area smaller than or equal to 0.5 µm^2^. Bouton sizes ≥ 0.3 µm in diameter are discernible on axons (Innocenti and Caminiti 2017), and the boutons of the CRc axon plexus were quite large. An envelope of 0.5 µm radial distance was generated around the blood vessel. Every sphere located within that distance of ≤ 0.5 µm to the vessel`s surface was counted and taken as potential interaction between vessel and bouton. Sphere numbers were plotted in relation to the length of the vessel segment assessed, normalized to 100 µm. As internal control, artificially generated tubes—virtual blood vessel segments—were generated in Imaris and embedded into the confocal stack at the same z-level near the genuine vessel segment of interest. An average tube diameter of 4.8 µm was selected (see Sobierajski et al. 2024). We hypothesized that a systematically higher proportion of close appositions of boutons to genuine blood vessels versus the artificial elements would suggest a preference of the CRc axons for the vessels. Graphs were made with SigmaPlot 12.3 (Systat Software GmbH).

## Results

### Developmental changes of the pig neocortex

**Fig. 1:**
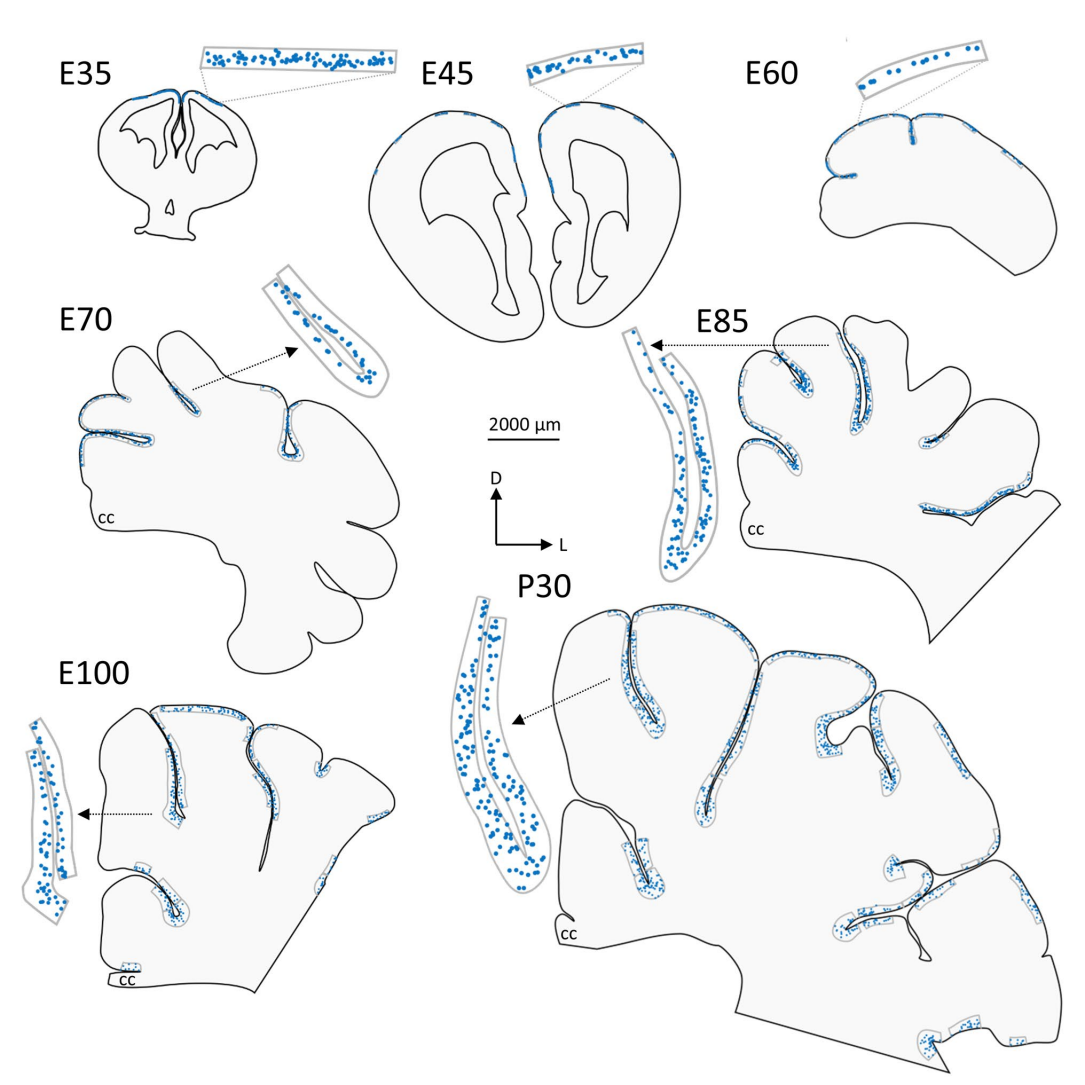
Development of the pig cortex. Neurolucida reconstructions of selected coronal sections (all paraffin material) from embryonic day (E) 35 to postnatal day (P) 30. Sections of E35 and E45, at a frontal level, have both hemispheres because the whole heads were embedded and sectioned. Sections at E70-P30 are dorsoparietal through the anterior part of the visual cortex; the E60 section is at a slightly more caudal level. The scale bar accounts for all sections. Note the enormous increase in cortical size and the increase in the depth of MZ/L1. A medial-to-lateral gradient was not apparent. Every blue dot in the MZ/L1 is a Reelin-positive soma with the nucleus in the plane of the section. The fields with blue dots were also used for the quantitative analysis. Representative fields are given at higher magnification. D, dorsal, and L, lateral for E60 to P30; CC, corpus callosum. Scale bar for all: 2 mm

The present study is restricted to the neocortex. We did neither examine the hippocampus nor the olfactory cortex ventral to the rhinal fissue. Sections analyzed were from frontal cortex (level of the hem), parietal cortex (with beginning of the dorsal hippocampus), and anterior visual cortex caudal to the ansate sulcus (Ernst et al. 2018) still at the level of the dorsal hippocampus. Figure 1 compiles representative coronal sections at the same magnification to provide a context to the changes of the Reelin-positive neuronal populations. It shows the size increase and the massive cortical expansion from E35 to P30, and the foliation beginning around E60. Selected regions of the MZ preferentially from sulcal flanks have been magnified and positioned next to each section. Each blue dot represents a Reelin-positive neuron irrespective of its size, shape or staining intensity, thus comprising Reelin-positive CRc and later on the Reelin-positive interneurons. Note the high density of cells at E35, the “dilution” already at E45, and clearly until E60/E70 suggesting that there is no substantial addition of CRc after E35. Note also the increased density of Reelin-positive cells at E85 which remained at that level until P30. This suggests a continuous addition of neurons, now presumably interneurons (see below), to balance the expansion of cortical surface and volume of L1.

**Fig. 2:**
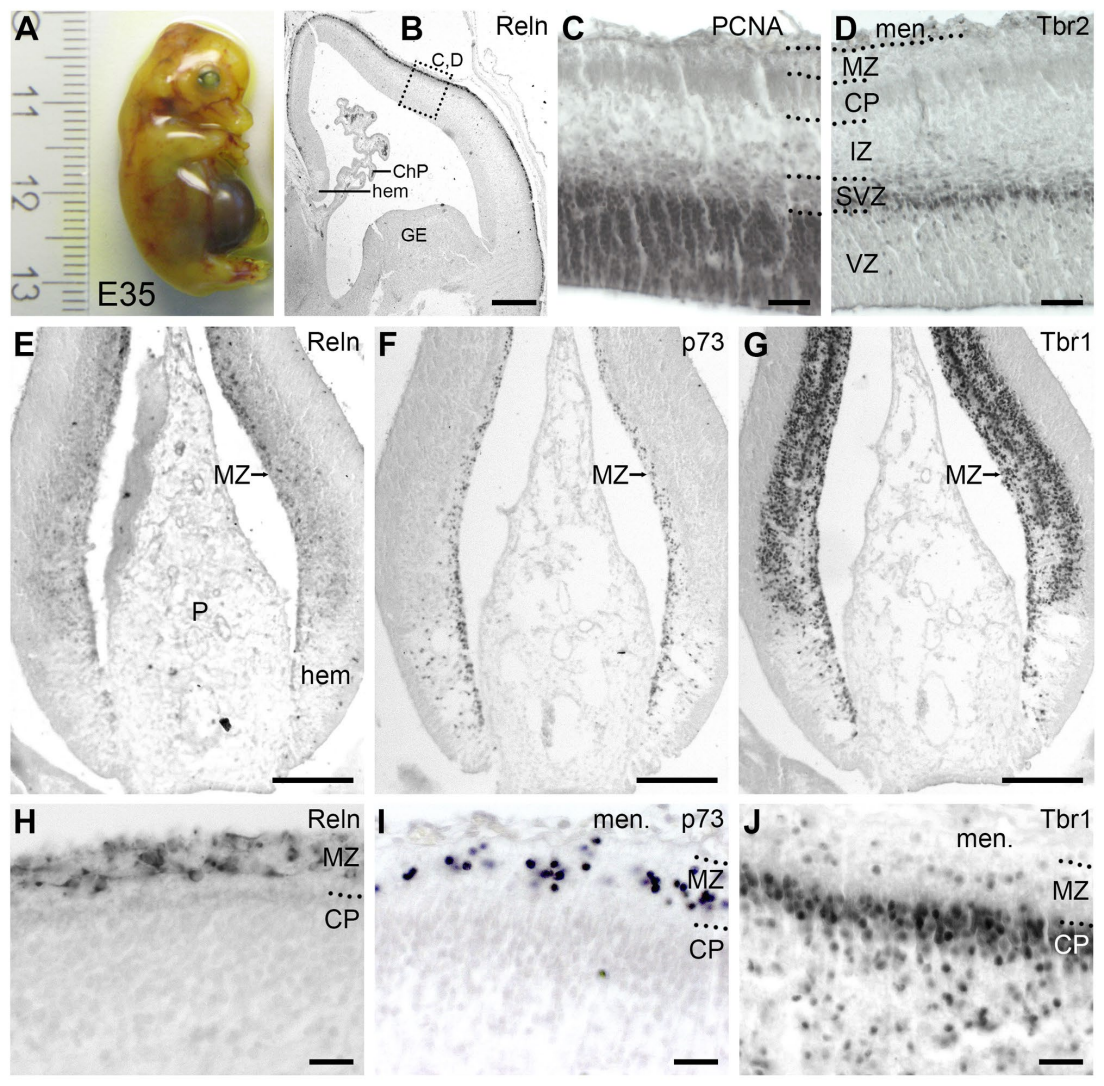
CRc in the cortex at E35.** A**. Photograph of an E35 fetus before paraffin embedding next to a scale [cm].** B**. Overview of a Reelin immunostained hemisphere. The cortical hem is indicated; note that CRc begin to express Reelin only when they reach the cortical marginal zone. The boxed area labeled C, D, indicates the position from which the photomicrographs in C and D have been taken from alternating sections. Cortical strata, including the incipient cortical plate, are laterally wider than medially.** C**. PCNA stains dividing cells in VZ and SVZ, but not MZ, CP and IZ. **D**. Tbr2 marks pallial intermediate progenitor cells in the SVZ, but not MZ. **E**. Reelin is not prominent in the cortical hem, here at a more rostral level than in B. Reelin expression becomes strong once CRc have reached the MZ. **F**. Nuclear p73 immunoreactivity in CRc born in the cortical hem from where they migrate into the neocortical MZ. **G**. Nuclear Tbr1 immunoreactivity in CRc born in the cortical hem. Cells continue to intensely express Tbr1 in the MZ. Early-born pallial cells of the incipient cortical layers are also Tbr1-positive. **H**. Reelin in the MZ at a more dorsal level. **I**. p73 in nuclei of CRc in the MZ. **J**. Tbr1 in nuclei in the MZ, CP and IZ. Dotted lines delineate laminar boundaries. Abbreviations: ChP, choroid plexus; CP, cortical plate; GE, ganglionic eminence; hem, cortical hem; IZ, intermediate zone; men., meninges; MZ, marginal zone; P: paraphysis; SVZ, subventricular zone; VZ, ventricular zone. Scale bars: 200 μm in B; 30 µm in C, D; 75 µm in E, F, G; 20 µm in H, I, J

The youngest stage E35 (Fig. 2A) was characterized by the presence of the cortical hem at the interface of choroid plexus *anlage* and telencephalon (Fig. 2B). Its human equivalent is Carnegie stage 21 at 8 gestational weeks (GW) (Meyer et al. 2000). The incipient cortical plate (CP) was still narrow (Fig. 2B, boxed area labeled C, D). Higher magnifications of alternating sections revealed a large VZ positive for PCNA (Fig. 2C) showing that cell proliferation was restricted to the proliferative VZ and SVZ. Tbr2 is a marker of pallial intermediate progenitor cells (Englund et al. 2005). Accordingly, Tbr2 was expressed only in the narrow subventricular zone (Fig. 2D). The pattern of PCNA and Tbr1 at E35 suggested that CRc do not divide locally in the pig MZ and had rather migrated tangentially from the cortical hem and possibly from other known sources of CRc.

At E45, the pig cortex is still lissencephalic (Fig. 1), with the exception of the hippocampal fissure that appears concurrent with the formation of the hippocampus. Based on the thickness of CP and intermediate zone, the human equivalent would be around 14–16 GW. E60 represents midgestation (birth in pig at 114 gestational days), and the first shallow sulci marked the start of a rapid process of foliation. At E70, the cortex displayed numerous sulci and gyri. At E85 an adult-like gyrification pattern was established, even though during the late gestational (E100) and the first postnatal month the size of the cortex and depth of the sulci continued to increase (Fig. 1) (see also Ernst et al. 2018, their Fig. 1). This is in contrast to the human brain, which at midgestation (about GW 20) is still smooth, and most intense foliation takes place in the last two months before birth (Chi et al. 1977).

### The lissencephalic stages E35 and E45

At E35, the distinctive structure was the cortical hem which gave rise to the large population of CRc all over the neocortical MZ. CRc markers are cytosolic Reelin (Fig. 2E), nuclear p73 (Fig. 2F) and nuclear Tbr1 (Fig. 2G). In fact, p73 is the only CRc marker not expressed in other cortical cell populations, and p73 was already seen in the deep cortical hem, as soon as CRc were born (Fig. 2F). Reelin and Tbr1 expression became stronger in CRc which had left the hem and aggregated in the MZ (Fig. 2E, G). In the MZ, the three markers were expressed by virtually all neurons. Also, the MZ was thin and entirely filled with CRc, which displayed an immature monopolar morphology, were arranged in several rows (Fig. 2H) and sometimes seemed to form clusters with no apparent gradient from dorsal to lateral at E35 (Fig. 2I). Tbr1 expressed by postmitotic pallial cells (Hevner et al. 2001, 2003) was also present in the incipient CP and intermediate zone (Fig. 2G, J).

**Fig. 3:**
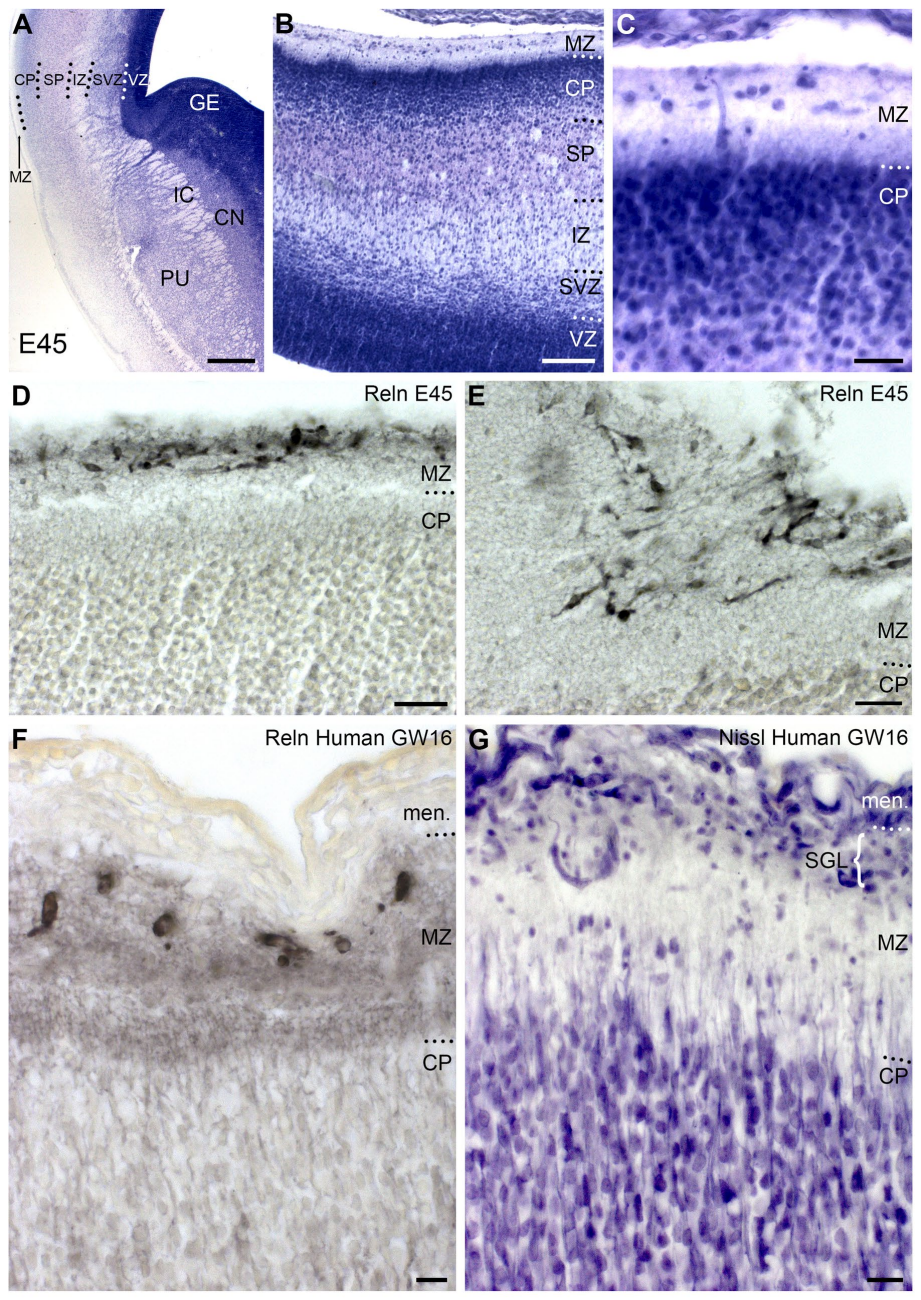
CRc in E45 pig cortex and human cortex at 16 GW. **A**-**C** Thionin-stained sections show the increasing differentiation of cortical strata and basal ganglia. **A** Section at the level of the basal ganglia. **B** The cortical strata with the MZ. **C** MZ and CP. **D** Reelin-positive CRc occupy the upper MZ. **E** CRc change orientation and aggregate at a shallow indentation of the dorsal cortex, possibly a first indication of an incipient sulcus. **F** The MZ of a human fetus at 16 GW. At this stage, CRc are located in the upper MZ, whereas their dense, intensely Reelin-positive axonal plexus occupies the lower half of the MZ to the border of the CP. **G**. Thionin staining of an alternating section. Human CRc are immersed in the subpial granular layer (SGL) directly beneath the meninges, which provide small descending blood vessels. Compared to human, the pig MZ (in C) lacks an SGL. Abbreviations: CN, caudate nucleus; IC, internal capsule; PU: putamen; other abbreviations as in Fig. 2. Scale bars: 250 μm in A; 100 μm in B; 30 μm in C, D; 20 µm in E; 10 μm in F, G

At E45, all cortical strata and the ganglionic eminences were more developed than at E35, and the internal capsule crossed the pallial/subpallial boundary (Fig. 3A). Also, the MZ was wider (Fig. 3B, C), and CRc were more dispersed, reflecting the expansion of the cortical surface. CRc occupied the outer MZ and were separated from the CP by a cell-sparse deep MZ. CRc had a more mature morphology compared to E35, extending one or two main dendrites mostly parallel to the pial surface (Fig. 3D). Remarkably, at a slight indentation of the dorsal pial surface, possibly a first sign of cortical folding, CRc became obliquely or even vertically orientation (Fig. 3E). Reelin expression was strong, however, a horizontal Reelin-positive axonal plexus was not visible in the deep MZ. This plexus is a prominent structure in the human MZ at the corresponding age of 16 GW (Fig. 3F). Another important difference between pig and human was the absence of the SGL of Ranke (Ranke 1910; Brun 1965), a dense aggregation of GABAergic, calretinin-positive granule cells characteristic of the fetal human cortex from 14 to 25 GW (Meyer and González-Gómez 2018a, b). The SGL is visible in Nissl-stained human sections (Fig. 3G), but in the E45 pig cortex there were very few small cells besides the CRc (Fig. 3C).

### The gyrated cortex after midgestation

**Fig. 4:**
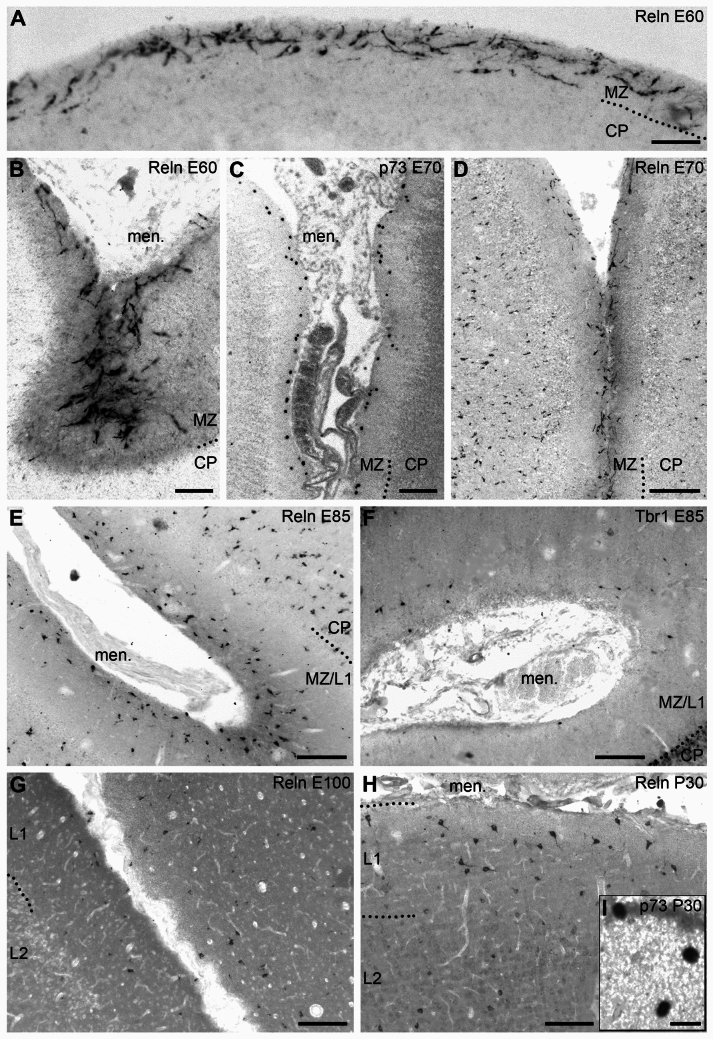
Development of CRc from E60 to P30. **A** E60; Reelin-positive CRc along a cortical convexity. Many CRc are still close to the pial surface and horizontally orientated, however, others adopt an oblique or vertical orientation and are more deeply positioned. There are no Reelin-positive interneurons in the superficial CP yet. **B** E60; numerous oblique and vertically oriented Reelin-positive CRC in a small sulcus. In the sulcal depth, the neuropil of the MZ shows somewhat stronger Reelin immunoreactivity. **C** E70; p73 in CRc in the MZ of a major sulcus occupied by meninges and meningeal vessels (which in part show some unspecific staining). The labeled nuclei may occur quite regularly spaced (left flank) or cluster in small groups. Most nuclei were close to the pia. **D** E70; Reelin-positive CRc in a sulcus. Compared to E60 (A), the CRc are less densely arranged. Reelin-positive interneurons are present in the cortical layers with a certain intensity gradient with the weakest labeled cells in the upper CP and lower half of the MZ.** E** E85; high density of Reelin-positive neurons, many of which are interneurons, while CRc have decreased in number. Reelin-positive interneurons were now common in L2 and also in the MZ/L1. They appear more differentiated and express higher levels of Reelin compared to E60. **F** E85; Tbr1 in a sulcus. There are much fewer CRc than at E65 suggesting that most of the Reelin-positive cells in E were indeed interneurons. **G** E100; the density of Reelin-positive cells appears a bit lower due to the fact that many cells are weakly labeled. CRc were rare. **H** P30; multipolar Reelin-positive interneurons scattering in L1 and L2. The distribution is similar to that at E100 suggesting that the adult-like pattern is already achieved before birth. **I** Inset in **H** shows three CRc identified by nuclear p73 immunoreactivity in layer 1 at P30. Isolated cells or small groups like this one found in the temporal lobe are occasionally present, albeit in an apparently random fashion. All sections cut at coronal plane around the representative sections plotted in Fig. 1. Scale bars: 30 µm in A, B; 70 μm in C; 100 µm in D, E, F, G, H; 10 µm in I. Abbreviations as in Fig. 2

At E60, the first sulci were observed (Fig. 1; Supplemental Fig. S1A-D). The PCNA-positive proliferative SVZ (Supplemental Fig. S1A) was highly developed underlying a broad gyrus and compressed below the sulcus. Similarly, the subplate was narrow below the sulcus (the zone with scattered Tbr1-positive nuclei in Supplemental Fig. S1B; and the clear zone in Supplemental Fig. S1C). In the sulcus, vimentin-positive radial glia fibers converged upon the CRc (Supplemental Fig. S1C, S1D) visualized with Tbr-1 (Supplemental Fig. S1B). E60 and E70 were the stages when Reelin expression in CRc was most prominent over the entire cortex (Fig. 4A), in particular near the bottom of the sulci, where the neuropil of the entire MZ was intensely positive (Fig. 4B). CRc were monopolar or had dendrites from the two somatic poles. Dendrites showed a higher degree of differentiation, albeit without displaying immunopositivity in thin side branches. Cells were horizontally oriented along gyral apex and sulcal flanks, although along the sulcal flanks, they also adopted oblique orientations, and they frequently became perpendicular to the pial surface at the sulcal bottom (Fig. 4B). At E70, most p73 nuclei resided near the pial surface of the now much wider MZ (Fig. 4C). The pattern was mirrored by the Reelin staining (Fig. 4D), and the basic morphology of the cells was the same as at E45. Signs of regression or degeneration, such as vacuoles in the cytoplasm, were subtle or absent. Further, at E70, Reelin-positive interneurons had appeared in the layers of the cortex, while the lower MZ was still largely void of Reelin-positive neurons.

**Fig. 5:**
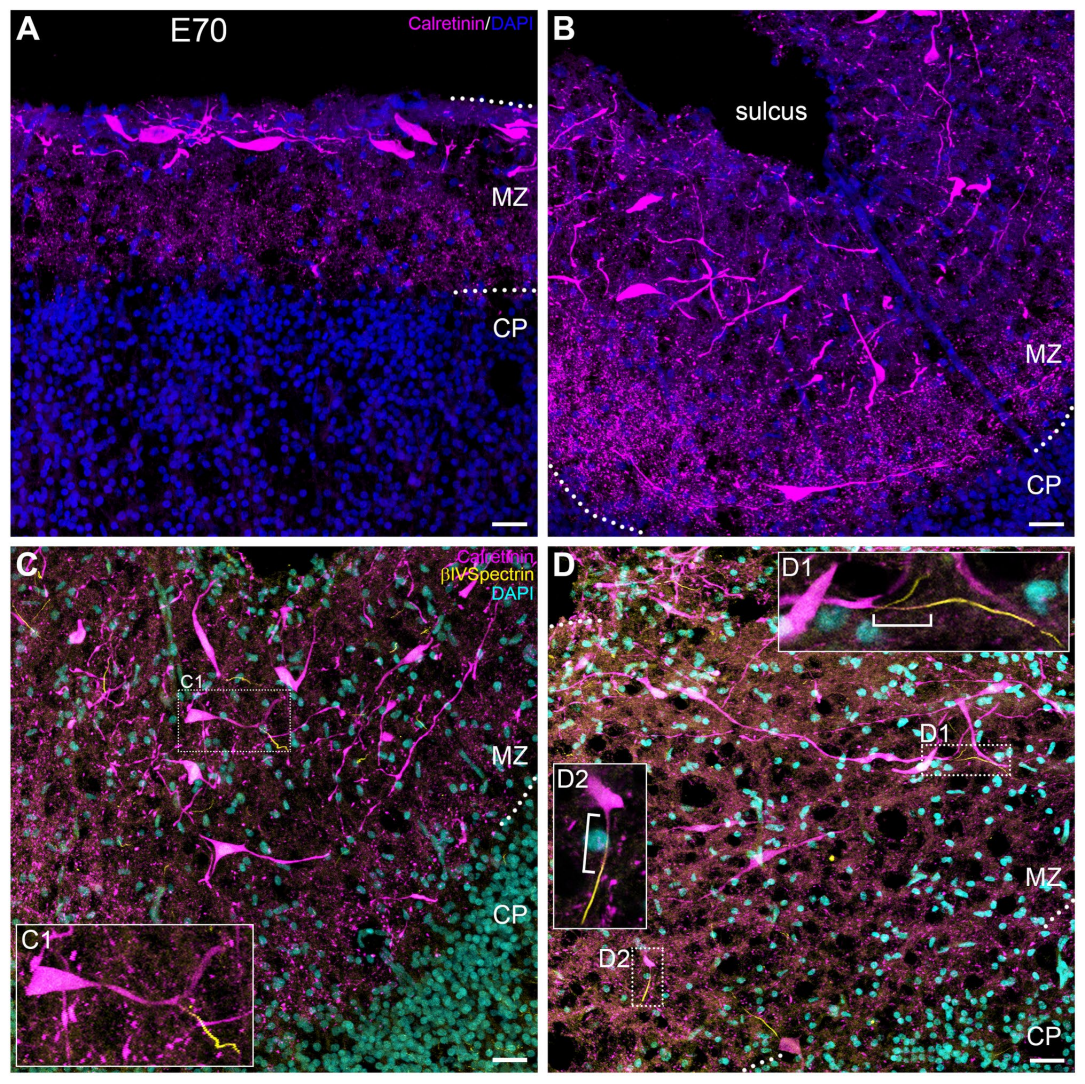
Calretinin staining at E70 and identification of axons. **A** Elongated mostly bipolar horizontally oriented CRc in the upper half of the MZ at an apex of a gyrus. Neither calretinin nor Reelin labels the fine filamentous appendages which commonly characterize the Golgi- or lipophilic dye-stained CRc.** B** CRc at the bottom of a sulcus with horizontal and vertical orientations. Cells mostly occupy the upper half of the MZ, and a varicose axonal plexus fills the lower half. **C**, **D** Axons are unequivocally identified by their βIV-spectrin-positive AIS. Note that axons emerge from thicker dendritic segments which may be not always connected to a parent soma as in insets C1 and D1, D2. Note also that the βIV-spectrin-positive domain always begins after a gap of variable length as indicated by the white brackets in insets D1, D2. Scale bars: 25 µm in **A**-**D**. Abbreviations as in Fig. 2

Reelin-positive pig CRc did not display the highly ramified axonal and dendritic trees so prominent in the human cortex at midgestation. Typically, the CRc axon plexus in the lower half of the MZ was Reelin-positive in the human, but not so in pig. We therefore employed the established CRc marker calretinin at E70 which is before the accumulation of interneurons in the MZ in sections equivalent to those shown in Fig. 1. Using 35 µm thick frozen sections we found intensely calretinin-positive CRc which displayed the same features observed with Reelin staining. In addition, calretinin staining visualized thin processes projecting into a varicose plexus filling the lower MZ to the boundary of the CP at an apex of a gyrus (Fig. 5A) and even more dense at the bottom of a sulcus (Fig. 5B). The varicose bouton-rich plexus resembled that reported for perinatal mouse cortex (Anstötz et al. 2014). CRc respond to major neurotransmitters and generate action potentials (Kirischuk et al. 2014, for review). Yet, it was not possible to unequivocally address thin processes emerging from the CRc as axons. To confirm the axonal nature, we probed for the presence of the axon initial segment (AIS), the molecularly unique axon-specific domain necessary for generating action potentials. In mouse cortex at E14.5, an age roughly comparable to pig E70, CRc display ankyrinG-positive AIS (Gutzmann et al. 2014). The protein βIV-spectrin is another established marker for the AIS and indeed, it revealed that pig CRc at E70 had axons frequently emerging from their long dendrites and less often from the somata. The βIV-spectrin-positive AIS always started after a substantial gap of 10–20 µm from the point of origin (Fig. 5C, D; Supplemental Fig. S2A, B, C). Most AIS ended abruptly when the axons left the sectional plane. For the longest AIS preserved within a section and the confocal stack, respectively, we determined lengths between 40–60 µm. This was substantially longer than the AIS of pyramidal cells of the cortical layers (Supplemental Fig. S2D) of about 20–30 µm at E70 (Ernst et al. 2018).

### The switch to the adult-like cellular composition in MZ

The E85 brain displayed an almost adult-like gyration pattern (Fig. 1). Layers were well defined in Nissl sections (Ernst et al. 2018) suggesting that the migration period was largely completed. This was reflected by a change in cell populations in the MZ/L1. It contained a high density of Reelin-positive neurons still more aggregated in the upper but also scattering in the lower MZ (Fig. 4E). Many of them were not CRc as demonstrated in Fig. 4F, where a similar sulcus was immunostained with Tbr1 which is not expressed in interneurons. Compared to E70, there were not many cells remaining in the Tbr1 staining, and with the typical CRc-like morphology in Reelin staining. However, large interneurons in layer 1 were difficult to distinguish based on morphology only (see Fig. 7). Thus, from E85 onwards, interneurons became an important source of Reelin in layer 1 and in the cortical layers. In perinatal cortex at E100 (Fig. 4G) and at P30 (Fig. 4H), the density of Reelin-positive interneurons appeared lower than at E85. Yet, this impression was attributable to a much weaker Reelin staining intensity of the L1 interneurons compared to CRc. The plots done at high magnification (Fig. 1) revealed a rather constant density of labeled neurons suggesting that interneurons now dominate the L1 cell population. CRc were, however, not completely absent after birth (Abraham et al. 2005). Intermingled with the often multipolar interneurons at P30 we observed occasional CRc as confirmed by the most specific CRc marker p73. Sometimes they occur in small groups of 2 or 3 cells, which apparently had found some niches for survival (Fig. 4I). Their presence seemed random and not bound to specific locations.

### Quantitative analysis of Reelin-positive neurons in MZ and cortical layers

**Fig. 6:**
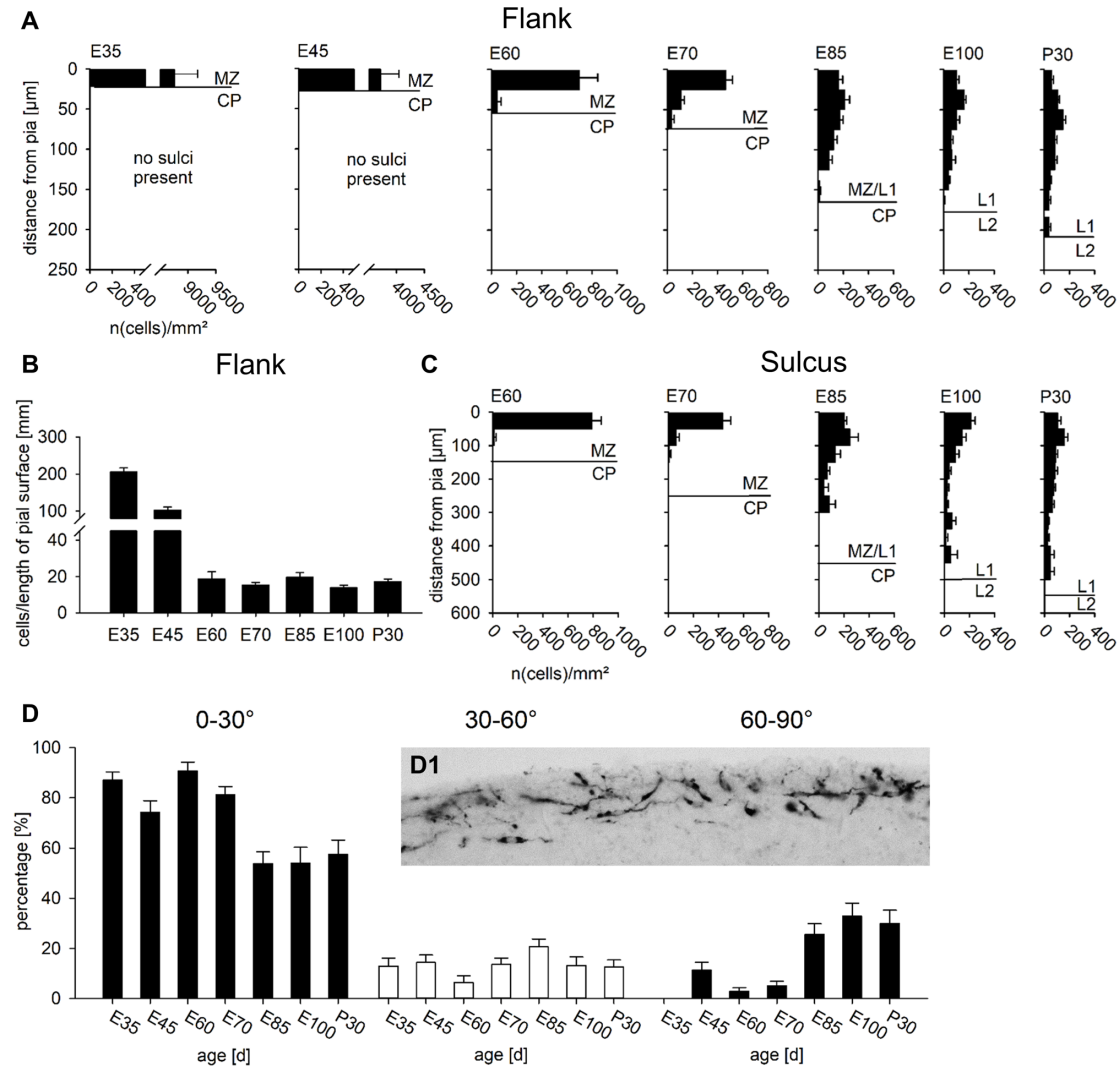
Quantification of Reelin-positive cells in the MZ/L1. **A** The number of Reelin-positive cells [mm^2^] of the MZ/L1 determined in dorsal aspects of the lissencephalic cortex at E35 and E45 and at the flanks of the forming gyri at E60 to P30. The position of the cells has been sampled in relation to their depth from the pial surface. With migration nearly completed at E85, we address the MZ from E100 onwards as Layer 1 (L1). **B** The number of Reelin-positive cells per millimeter cortical surface length at the sulcal flanks demonstrates the highest densities at E35 and E45 and rather constant lower numbers thereafter. **C** After the onset of foliation at E60 the density of Reelin-positive cells was determined in sulci, also to demonstrate the substantial increase of the width of the MZ/L1 to about 500 µm at the bottom of the sulci, independently from the rostrocaudal level. **D** Percentage of Reelin-positive cells in the MZ/L1 determined at apex (inset) and flanks of gyri which have the longest soma axis 0–30° near-horizontal, 30–60° oblique, or 60–90° more perpendicular to the surface. The assessment has been done from paraffin-embedded material sectioned to 10 µm thickness in 25 µm bins at E35 and E45, and in 50 µm bins from E60 onwards. Bar graphs report the mean with S.E.M.. Abbreviations as in Fig. 2

The qualitative data were mirrored by counting Reelin-positive somata per mm^2^ of the MZ/L1 in relation to their depth from the pial surface (Fig. 6) using our series or paraffin-embedded sections (Fig. 1). The plots revealed a narrow MZ of only 25–30 µm depth at E35 densely packed with Reelin-positive somata. The approach normalizing to the area [mm^2^] of the MZ yielded thousands of cells. More realistic, expressing cell numbers per mm length of the pial surface (Fig. 6B) yielded ~ 200 Reelin-positive somata. Already by E45 the density per area and per surface length was reduced due to dilution in the expanding volume. At E60, only about 20 labeled cells per mm surface length were present and remained from E70 until P30. What resembled a plateau must be interpreted as a continuous addition and differentiation of Reelin-positive interneurons when considering the substantial increase in cortical volume and surface. At E60, the MZ was clearly wider at flanks (Fig. 6A) and even more so at the depth of the sulci (Fig. 6C). Until E60 the vast majority of somata were in the outer half of the MZ/L1, the typical position for CRc. Reelin-positive interneurons started to populate the infragranular cortical layers still leaving the CP at E60 void of labeled cells (Supplemental Fig. S3). At E70, the first Reelin-positive interneurons had appeared in the lower half of the MZ/L1. At E85, the MZ reached about 200 µm width at the flanks and about 400 µm width in the sulci, almost the dimensions seen at P30. From E85 to P30, Reelin-positive cells distribute throughout the MZ/L1 and L1, still with some preference for a position in the upper half. Further, all cortical layers were now occupied by Reelin-positive interneurons (Supplemental Fig. S3).

The switch between E60 to E85 was also reflected by the changing orientation of the Reelin-positive somata determined at flanks. Until E70 most somata have the longest axis within 0–30° parallel to the pial surface (Fig. 6D, inset D1). From E85 onwards a substantial fraction had the longest axis more perpendicular to the pial surface (Fig. 6D). Together, the change in density, in position, and in orientation supported the observation that the switch of cell classes proceeds mainly between E70 and E85.

### Reelin in excitatory CRc versus Reelin in inhibitory interneurons in the MZ

**Fig. 7:**
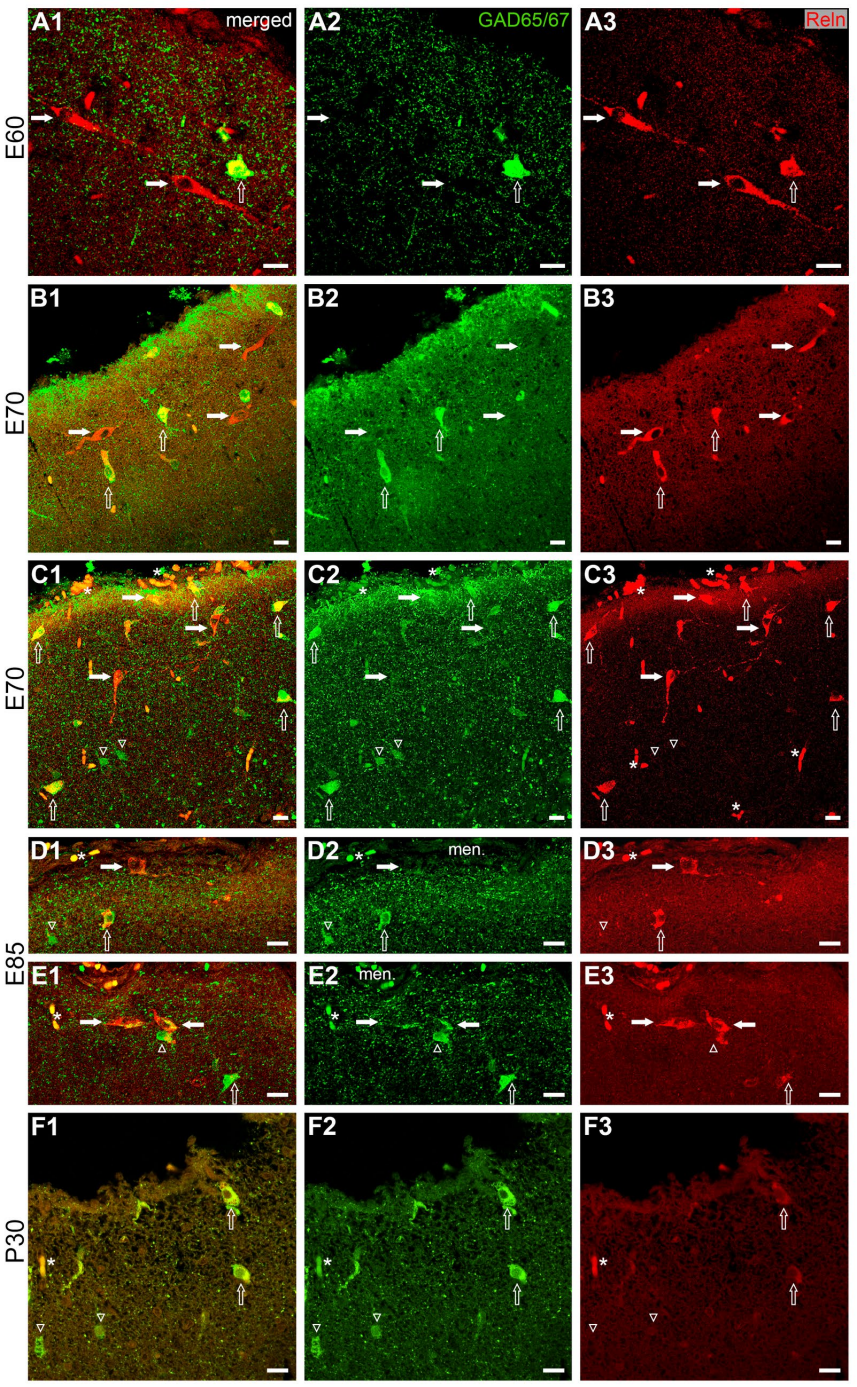
Expression of Reelin and GAD from E60 to P30. **A1**-**3**. E60. Two horizontal CRc are indicated. A superficial interneuron co-expresses Reelin and GAD. **B1**-**3**. E70, at a flank of a parietal gyrus. Three horizontal CRc. Interneurons are now more common, with various intensities of Reelin or GAD immunoreactivity and variable sizes. **C1**-**3**. E70 at the bottom of a sulcus. CRc with horizontal and vertical orientation. Note that large vertical or horizontal neurons may be interpreted as CRc due to Reelin expression, but co-expression of GAD reveals their interneuronal nature. Other GAD-positive interneurons are negative for Reelin.** D1**-**3**. E85 at a flank. One CRc next to GAD-positive and double-labeled interneurons.** E1**-**3**. E85 near a sulcus. Two CRc next to GAD-positive and double-labeled interneurons. Note in **D**,** E** that the MZ/L1 is filled densely with GAD-positive axonal terminals which also cluster around the CRc somata. **F1**-**3**. P30 at a flank. Double-labeled and GAD-only interneurons dominate the MZ. White symbols: Reelin-only CRc marked by solid horizontal arrows; GAD/Reelin-positive interneurons marked by open arrows; GAD-only interneurons marked by open arrowheads. Small asterisks mark unspecific staining e.g. in blood vessels. Abbreviations as in Fig. 2. Scale bars: 10 µm in A-F

CRc are pallial excitatory neurons (del Río et al. 1995; Hevner et al. 2003). A way to distinguish CRc from inhibitory interneurons is by double-staining for Reelin and glutamate decarboxylase (GAD) (Fig. 7). The earliest successful co-labeling was obtained at E60, when CRc were the dominant cell type in the MZ. A few quite large interneurons were observed co-expressing both proteins (Fig. 7A1–3). At E70, the main orientation of CRc varied according to their position in sulci and gyri. At apex or flanks (Fig. 7B1–3), CRc had a mostly horizontal or oblique orientation, while at the bottom of a sulcus vertical and often deep forms predominated (Fig. 7C1–3). However, some large vertical or horizontal CRc-like Reelin-positive neurons co-expressed GAD and were identified as interneurons. Thus, at E70, it became evident that interneurons in the MZ did not constitute a uniform cell class since they displayed various sizes and shapes and co-expressed Reelin at variable levels of intensity while others were Reelin-negative. At E85 (Fig. 7D1–3, E1–3) CRc were still occasionally present (Figs. 7D, E, 4F) intermingled with double-labeled or GAD-only interneurons. Reelin-expressing interneurons of the cortical layers largely belong to the non-fast-spiking types including Martinotti neurons which project into the MZ/L1. Martinotti cell projections and axons of local MZ/L1 interneurons plus the ascending afferents from subplate and zona incerta (Kirischuk et al. 2014) contributed to the dense GAD-positive axonal plexus and the boutons surrounding CRc at 70 and E85 (Fig. 7D1, E1). At P30 (Fig. 7F1–3) most of the cells were Reelin-positive interneurons or expressed only GAD. We may summarize that after midgestation, CRc gradually become sparse in the MZ whereas interneurons are taking over and remain into postnatal life.

### The relation of the CRc axon plexus to blood vessels

The lower MZ is the place where blood vessels branch before descending further in the gray matter (Sobierajski et al. 2024). Further, the density of blood vessels trunks is high at the bottom of sulci where also the density of CRc is higher than at gyral flanks or apices. Nitric oxide generated by CRc (Judas et al. 1999; Meyer and González-Gómez 2018a) could influence on blood vessels. Using tomato lectin to label vessels and calretinin for the CRc axon plexus we probed for a potential preference. As a control structure, an artifically generated tube with the dimensions of MZ blood vessels (Sobierajski et al. 2024) has been virtually embedded into the confocal stack of the axon plexus near to and at the same z-level as each tomato lectin-positive genuine vessel segment selected for assessment (Supplemental Fig. S4A, B). Counting the number of close appositions of calretinin-positive boutons within a virtual envelope of 0.5 µm radial distance from the surface of the genuine and the virtual blood vessels revealed a substantial variability of the number of appositions per segment length (almost factor of 3) but no statistical difference between genuine and virtual vessels (Supplemental Fig. S4C).

## Discussion

### The timeline of Reelin expression in pig

The emergence and developmental history of Reelin expression in the MZ of the pig follows a chronological pattern also observed in other mammalian species. In rodent and human, CRc are the first neurons that appear in the embryonic cortex. CRc express high levels of Reelin, are transient, and disappear by cell death at a certain developmental stage (Marín-Padilla, 1998; Meyer and Goffinet 1998; Meyer et al. 2000; Derer and Derer 1990; del Río et al. 1995, Elorriaga et al. 2023, Meyer and González‐Hernández,1993). In the pig, a precocial species that has an almost mature, functional cortex at birth, the decline of CRc began shortly after midgestation. However, we did not observe clear signs of degeneration, and CRc were still occasionally present after birth. Dilution in an expanding cortex may thus be an additional factor (see also Abraham et al. 2005), and more likely than morphological remodeling. By contrast, in rat and mouse, altricial species which require an extensive postnatal weaning period, CRc die during the second postnatal week (del Río et al. 1995; Derer and Derer 1990). In human, another semi-altricial species, the situation is more complex but eventually, early appearing, intensely Reelin-positive human CRc also die during the second half of gestation (Meyer and González‐Hernández, 1993; Meyer and González-Gómez 2018a, b). In parallel with the demise of CRc, Reelin-positive interneurons appear in the mammalian MZ and also in the cortical layers, and they persist into adult life.

In the earliest stage available in our study, at E35, CRc were still seen to emerge from the cortical hem which is the main origin of p73-positive neocortical CRc from where CRc migrate along complex tangential routes. However, they were already quite numerous in the MZ of the entire neocortical anlage. This suggests that the first CRc had been generated even earlier. The initial E35 population seemed to constitute the main population, without substantial further additions from other possible sites such as the septum or the thalamic eminence, because from E35 onwards the density of CRc in the pig MZ steadily declined. From E35 to E60, the period of maximum cortical migration, CRc differentiated and emitted long processes, leading to a large amount of Reelin-positive immunoreactive structures at midgestation. The intense positivity of Reelin in CRc is due to the activating role of Tbr1 on Reelin expression (Hevner et al. 2003) and reflects the importance of CRc in radial migration.

In postnatal and adult cortex, Reelin is involved in synaptic plasticity and learning, which might be significant for the young piglet and its adaptation to the wilderness. Reelin influences on plasticity through binding to its receptor ApoER2, which, via differential splicing of exon 19, forms a functional complex with NMDA receptors in the postsynaptic densities of excitatory synapses. This way, Reelin modulates NMDA receptor activity, synaptic transmission, enhancement of hippocampal long-term potentiation, and memory (Beffert et al. 2005). Hitherto the role of Reelin in interneurons of the L1 had remained enigmatic. Conditional knockout mice with induced postnatal Reelin deficiency suggested that Reelin acts as “stop growth signal” restricting postnatal interneuron dendritic growth (Hamad et al. 2021).

### Comparative aspects of CRc in mammalian species

Our timeline of Reelin expression matches to a study of Reelin mRNA and protein expression in the developing domestic pig (Nielsen et al. 2010). Using Western blot analysis, Nielsen et al. detect the earliest Reelin protein expression at the preplate stage E28, an expression peak at E60, followed by a decrease. A qRT-PCR analysis shows very low levels of Reelin mRNA already at E21 that increases to peak at E60, declines to a plateau level to E100, and declines further to E115. This sequence of Reelin mRNA and protein expression fits in with our immunohistochemical results supporting the view that the generation of CRc starts well before E35. Reelin mRNA at E21 and Reelin protein by E28 suggest that CRc in pig are present at the preplate stage. Reelin-positive neurons in the MZ at E35 clearly demonstrate that CRc are present as early as the initial cortical plate stage albeit still with immature morphology. Assuming a generation and migration of CRc already at a preplate stage likely explains our observation at E35 that there was no apparent medial-to-lateral or rostral-to-caudal gradient in distribution of CRc. This assumption also makes the quantification (Fig. 7) of CRc numbers in the MZ and cell orientation preference reliable.

We conclude that there are no obvious differences in the prenatal development of the domestic pig and its counterpart, the wild boar, making our comparison one of the first studies which have analyzed an important aspect of cortical development between domestic and wild species. Furthermore, very few studies systematically examined mammalian CRc across species from their first appearance to postnatal life. The mouse was studied by del Río et al. (1995), and the rat by Meyer et al. (1998). Most current studies in transgenic mice are limited to the prenatal period, although the cortex matures principally after birth. Rodent CRc display a simple, mostly monopolar morphology parallel to the pial surface, plus fine processes ascending to the pia, visible in thick Golgi sections (Retzius 1893; Cajal 1980), after DiI injections (Meyer and González-Hernández 1993; Merkulyeva and Mikhalkin 2021), or with intracellular biocytin injections (Radnikow et al. 2002). In rodent, axons emerge from the soma opposite to the major dendrite (Anstötz et al. 2014).

The classical drawings depict CRc of dog, cat and lagomorph (Retzius 1893, 1894; Meyer and Ferres-Torres 1984; Merkulyeva and Mikhalkin 2021; Cajal 1891). Remarkably, in particular the lagomorph CRc resemble the pig morphotype very closely. In these orders the dendritic tree is predominantly bipolar, and the dendrites can split into long branches. The axonal origin is often from a dendritic branch far away from the soma (Cajal 1891) as now demonstrated for pig CRc. Notably, the axonal plexus visualized with calretinin was diffuse around the CRc and formed varicosities in the deeper half of the MZ. This is similar to the CRc axon plexus in rodents (Radnikow et al. 2002).

In pig, a single, initially quite large CRc population was gradually diluted until midgestation and then almost completely disappeared (Figs. 1, 6). In human, CRc appear gradually in several sequential populations (Meyer and González-Hernández 1993; Meyer and Goffinet; 1998; Meyer and González-Gómez 2018a, b). In the embryonic and early fetal human stages, CRc display the immature monopolar morphology possibly reflecting their migratory nature. As they mature around 15–16 GW, CRc adopt a variety of shapes, many of which are vertically oriented and emit a large number of dendritic side branches. Human CRc give rise to a dense horizontal Reelin-positive axonal plexus that occupies the lower half of the MZ. The plexus forms a veritable physical barrier between the MZ and the superficial CP. After midgestation, CRc degenerate and die, and the dense axonal plexus breaks down (Meyer and González- Hernández, 1993; Meyer et al. 2002; Meyer and González-Gómez 2018a, b). A population of smaller Reelin/p73/Tbr1/calretinin-positive CRc appear in the perinatal period in human mainly in a subpial position along the walls of later appearing secondary and tertiary folds, and often close to blood vessels (Meyer and González-Gómez 2018a, b). Gyrification in the pig cortex is basically established around E85, and although sulci still deepen later in development, the basic pattern is maintained into adulthood (Ernst et al. 2018) with secondary and tertiary sulci being not as prominent as in human. Accordingly, late-appearing CRc were not present in the pig. Human CRc cells thus clearly differ from the CRc of non-primate species, both in their more complex morphology and developmental history, reflecting a longer gestation period and an increasing complexity of cortical architectonics and functions.

Another difference to primate is the lack of an SGL. The human SGL contains CRc progenitors that provide additional CRc as the cortex grows. Further, the SGL harbors small GABA-ergic cells which provide a dense innervation to the transient CRc population and which – via GABA signaling—may be involved in their death (Meyer and Goffinet 1998; Meyer and González-Gómez 2018a, b). Also in pig, the density of GAD-positive axonal boutons increased in the MZ at E70 and E85, and concurrent with the decline of CRc. Thus, with the lack of an SGL, pig resembles all non-primates studied so far in that there is no indication for late-appearing CRc.

### The axons of pig CRc

A novel aspect is the unequivocal identification of the axon using the unique axon-specific molecular signature, the βIV-spectrin-positive AIS. Variation of AIS length and position close to or more distant to the soma can influence the intrinsic excitability of the neuron (Yamada and Kuba 2016). In some neuron types those with a long AIS close to the soma display a higher excitability (Gulledge and Bravo 2016; Chand et al. 2015). Also, modeling studies suggest that for large neurons a long AIS argues for a higher excitability. Pig CRc were indeed large neurons with axons from dendrites and long AIS of up to 60 µm, much longer than AIS of fetal pig cortical pyramidal cells at the same age (Ernst et al. 2018). These features resemble those reported for tonically active midbrain dopaminergic neurons which also have distantly emerging axons with up to 60 µm long AIS. Functionally, in these neurons a negative correlation has been reported between distance from the soma and action potential frequency whereas the length of the AIS correlates positively with action potential frequency (González-Cabrera et al. 2017; Meza et al. 2018; López-Jury et al. 2018). However, long AIS are not a general feature of early-born cortical neuron types. Entirely opposite, non-GABAergic neuropeptide Y-positive axonal loop neurons of the pig subplate at E70 display rather short AIS (Ernst et al. 2018). Further, most axonal loop subplate neurons in pig have axons from the somata similar to axonal loop neurons of postnatal cat cortex (Wahle et al. 2022) whereas pig CRc had axons frequently from a dendrite. In sum, CRc in pig MZ present relatively large mostly bipolar neurons with thick horizontal dendrites, frequently with distantly emerging axons, and a substantial gap before a long AIS. We can only speculate on the function. The excitability of CRc may vary across species and may also depend on the developmental time point, e.g. which neurochemical afferents have reached the MZ. Electrophysiology in slices of fetal pig cortex may unravel the differences between species or even between the orders of mammals.

### On the possible roles of CRc

Might CRc be involved in cortical gyrification and vasculogenesis? Some observations support this hypothesis, apart from the fact that human deficiencies of REELIN or TP73 lead to lissencephaly and mental retardation (Wallenmeier et al. 2021; Hong et al. 2000). Gyrification is a highly discussed, multifaceted process, and we refer here to the extensive review by Akula et al. (2023). CRc interact with and shape the radial glia (Soriano et al. 1997; Hartfuss et al. 2003) this way influencing radial migration. The subpial position of CRc and their tangential migration is regulated by CXCL12 in the meninges (Reiss et al. 2002; Tissir et al. 2004) via the CXCR4 receptor in CRc (Borrell and Marín, 2006; Paredes et al. 2006). Interestingly, p73 has been also implicated in vasculogenesis (Fernandez-Alonso et al. 2015), and p73-positive CRc around midgestation in pig and in perinatal human are closely adjacent to the pia and near the entrance of pial vessels. Vascular growth has to keep pace with the growing cortex. Matching to this, blood vessel formation in pig cortex commences at E45, and essential proteins of the blood–brain-barrier are present from E60 onwards (Sobierajski et al. 2024). In the lower MZ, CRc axons densely ramify around blood vessels entering from the pia and often branching before descending into the cortical plate. Further, the number of CRc and of blood vessels is always higher at the depth of the sulci, and sometimes it appears as if vessel segments are surrounded by boutons. Reelin is not vasogenic but nitric oxide via NADPH acts on vessels. Our analysis did not rule out the possibility that CRc could influence on blood vessel sprouting or branching via diffusible signaling factors. However, our assessment indicates that close contacts between boutons and vessels rather occur by chance and that CRc axons are not specifically targeted to blood vessels in the lower MZ.

We may summarize that even though cortex development of pig and human shares many common traits, their cell populations of the MZ/L1 are quite different, with the pig CRc resembling more the lagomorph than the human morphotypes.

## Supplementary Information

Below is the link to the electronic supplementary material.

Supplementary file1 (DOCX 2652 KB)

## Data Availability

All relevant data can be found within the article and its supplementary information. No datasets were generated or analysed during the current study.
