# Supplementary material for: Developmental changes of Reelin-expressing cell populations in the marginal zone of the neocortex of the European wild boar, Sus scrofa
Source: Brain Struct Funct. 2025 Jun 11;230(6):96. doi: 10.1007/s00429-025-02958-w (PMC12159104; doi:10.1007/s00429-025-02958-w)
Supplement: Supplementary file 1 — Supplementary file1 (DOCX 2652 KB) [file 429_2025_2958_MOESM1_ESM.docx]

**Supplement material** for

Developmental changes of Reelin-expressing cell populations in the marginal zone of the neocortex of the European wild boar, *Sus scrofa*.

Eric Sobierajski ^1*^, Miriam González-Gómez ^2, 3*^, Emilio González-Arnay ^2^, Petra Wahle ^1#^, Gundela Meyer ^3#^

* equal contribution

^#^ shared senior

^1^ Ruhr University Bochum, Faculty of Biology and Biotechnology, Developmental Neurobiology, 44870 Bochum, Germany

^2^ Institute of Neurocience, University of La Laguna, 38200, Santa Cruz de Tenerife, Tenerife, Spain.

^3^ Department of Basic Medical Science, Faculty of Medicine, University of La Laguna, 38200, Santa Cruz de Tenerife, Tenerife, Spain

Co-correspondence: [gundelam@aol.com](mailto:gundelam@aol.com), [petra.wahle@rub.de](mailto:petra.wahle@rub.de)


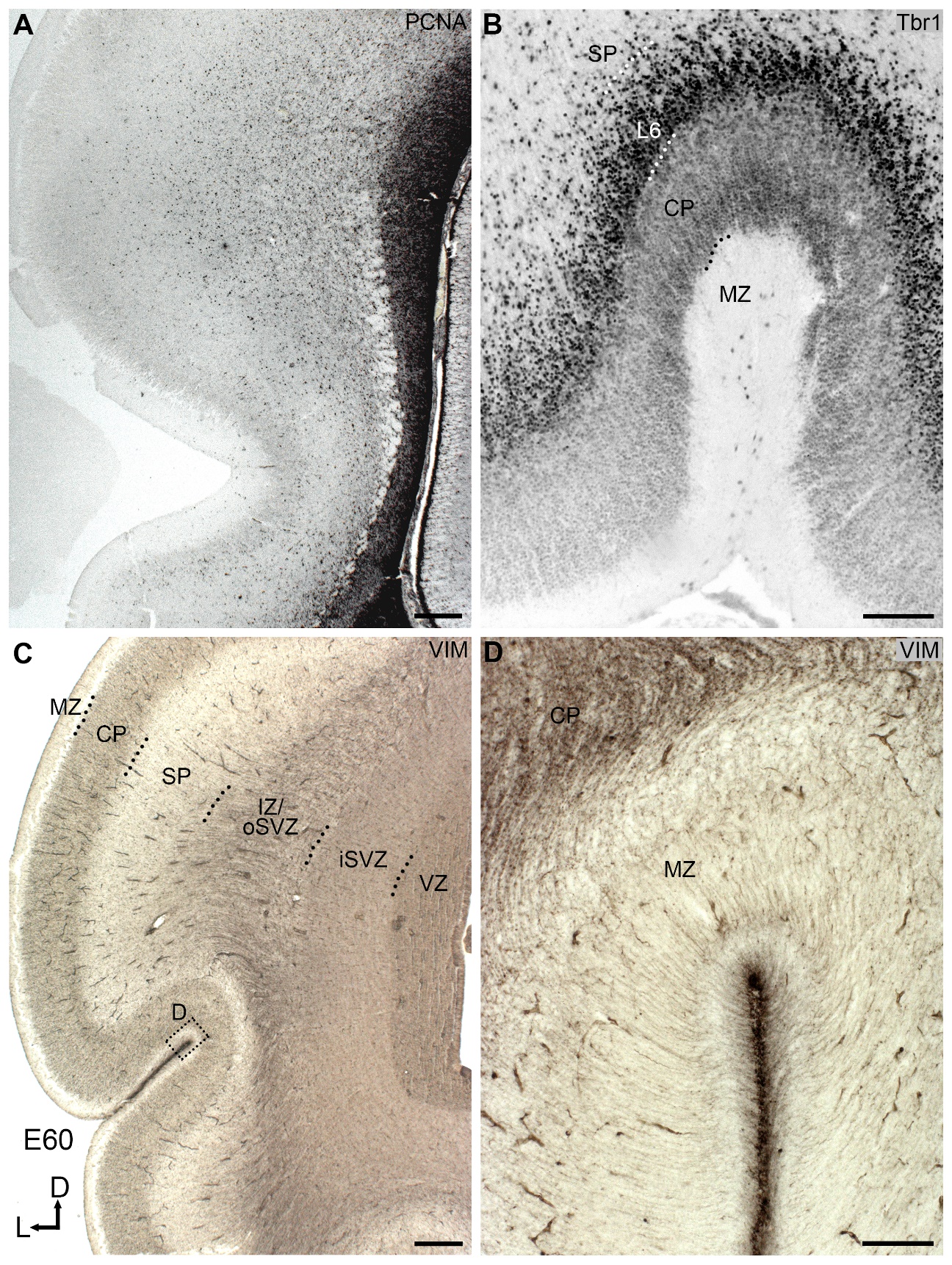


***Supplemental Fig. S1. The cortex at E60.***

*A. PCNA at a more caudal level shows the dividing cells in VZ, SVZ and even the lower IZ, although the exact boundaries have been difficult to delineate.*

*B. Tbr1 in CRc of the MZ and in cells of presumptive infragranular layers and subplate.*

*C. Vimentin-positive radial glia in the different cortical strata. Cortical plate (CP) and subplate (SP) are much wider compared to E45 but narrow at the bottom of the sulcus (boxed area).*

*D. The box indicated in C slightly rotated shows radial glia fibers converging upon the sulcus. The fibers extend their intensely positive endfeet in the zone corresponding to the pial surface. Scale bars: 100 µm in A, B, D; 400 μm in C. Abbreviations as in Fig. 2.*

*
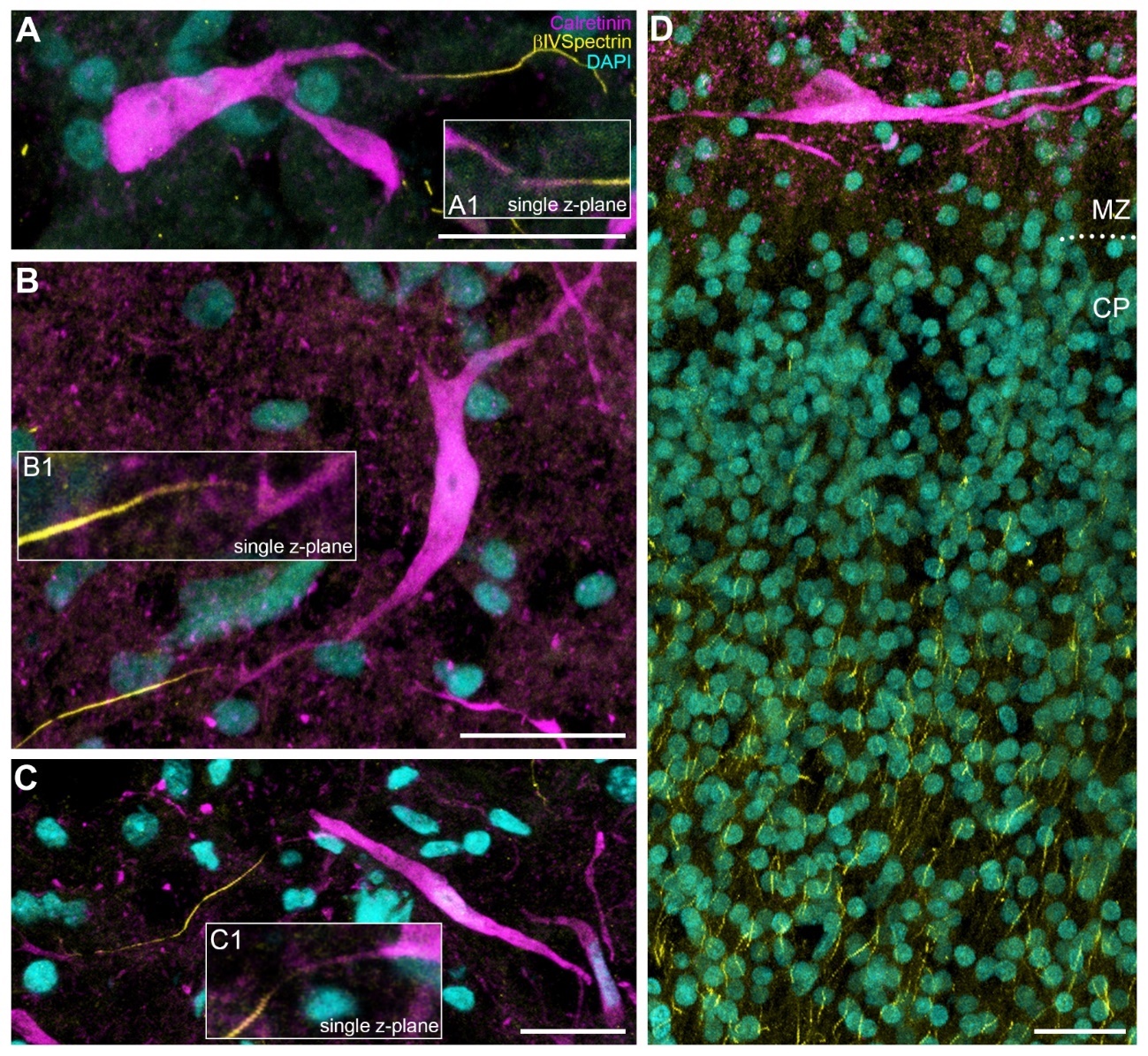
*

***Supplemental Fig. S2. Calretinin and βIV-spectrin-positive AIS at E70.***

*A, B, C. CRc with axons from dendrites. The nucleus with Calretinin-negative nucleolus is present in the elongated somata. Insets in B, C, are single t-planes showing the gap between the origin of the axon and the begin of the AIS at even higher magnification because the intensity of calretinin immunoreactivity tends to be low at the gap.*

*D. Overview of the lower half of the MZ with one deep CRc with horizontal dendritic branches and the adjacent CP. Note that the migrating neurons of the CP have not yet assembled a detectable βIV-spectrin-positive domain, only few short AIS were detectable. The more mature pyramidal cells of the presumptive infragranular layers below have AIS. Scale bars: 25 µm. Abbreviations as in Fig. 2.*


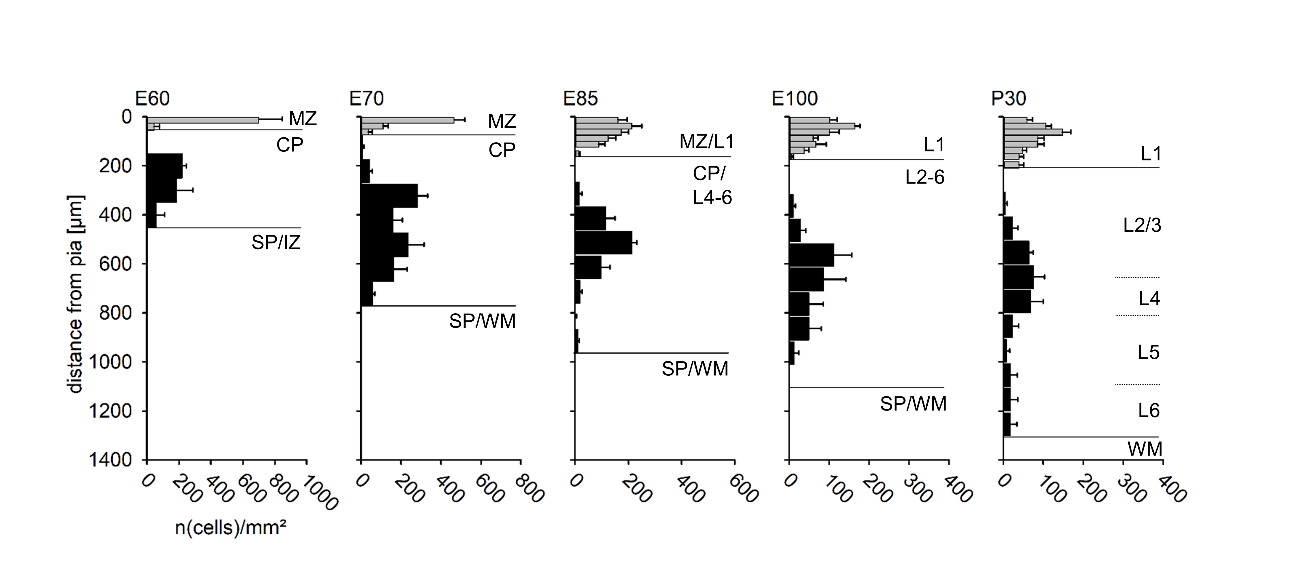


***Supplemental Fig. S3. Quantification of Reelin-positive cells in the cortical layers.*** *Substantial numbers of interneurons have reached the presumptive infragranular layers at E60. The cortex continuously widens and Reelin-positive interneurons populate all layers down to the white matter (WM) border with a certain enrichment in middle layer indicated for P30 from alternating thionin-stained sections. Cells have been scored in 100 µm bins from the WM border upwards along sulcal flanks not considering the apex and the depth of the sulci where layers become broader or variably compressed, respectively. In gray, the Reelin-positive cells of the MZ scored in 50 µm bins as given in Fig. 6. for direct comparison.*


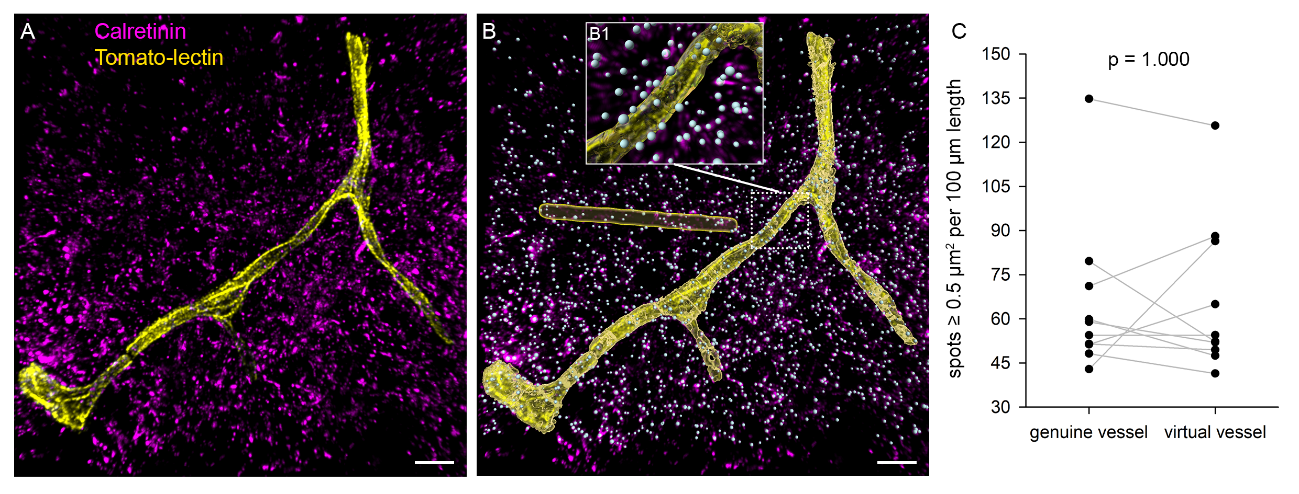


***Supplemental Fig. 4. The relation of the CRc axon plexus to blood vessels.*** *A. Representative tomato lectin-positive blood vessel in the lower half of the MZ (yellow) surrounded by calretinin-positive puncta (magenta). B. The 3D reconstruction. The inset B1 shows the spherical dots (former boutons) located around the vessel. The semitransparent tube is the virtual vessel embedded into the plexus near the genuine vessel of interest. C. Quantification of ≥0.5 µm^2^ boutons within the 0.5 µm thick envelope surrounding the genuine and the virtual vessel. Every dot represents the readout of one structure normalized to 100 µm length.*
